# PD‐1 expression is upregulated on adapted T cells in experimental autoimmune encephalomyelitis but is not required to maintain a hyporesponsive state

**DOI:** 10.1002/eji.201847868

**Published:** 2018-12-07

**Authors:** Iris Mair, Dario Besusso, Louise Saul, Sarju D. Patel, Rahul Ravindran, Rhoanne C. McPherson, Melanie D. Leech, Richard A. O'Connor, Stephen M. Anderton, Richard J. Mellanby

**Affiliations:** ^1^ MRC Centre for Inflammation Research Centre for Multiple Sclerosis Research Centre for Immunity, Infection and Evolution The University of Edinburgh Edinburgh UK; ^2^ The Royal (Dick) School of Veterinary Studies and The Roslin Institute, Division of Veterinary Clinical Studies The University of Edinburgh Hospital for Small Animals Easter Bush Veterinary Centre Roslin Midlothian UK

**Keywords:** Adaptation, Autoimmunity, Experimental autoimmune encephalomyelitis, PD‐1, T cell

## Abstract

T cell adaptation is an important peripheral tolerogenic process which ensures that the T cell population can respond effectively to pathogens but remains tolerant to self‐antigens. We probed the mechanisms of T cell adaptation using an experimental autoimmune encephalomyelitis (EAE) model in which the fate of autopathogenic T cells could be followed. We demonstrated that immunisation with a high dose of myelin basic protein (MBP) peptide and complete Freund's adjuvant failed to effectively initiate EAE, in contrast to low dose MBP peptide immunisation which readily induced disease. The proportion of autopathogenic CD4^+^ T cells in the central nervous system (CNS) of mice immunised with a high dose of MBP peptide was not significantly different to mice immunised with a low dose. However, autopathogenic T cells in mice immunised with high dose MBP peptide had an unresponsive phenotype in ex vivo recall assays. Importantly, whilst expression of PD‐1 was increased on adapted CD4^+^ T cells within the CNS, loss of PD‐1 function did not prevent the development of the unresponsive state. The lack of a role for PD‐1 in the acquisition of the adapted state stands in striking contrast to the reported functional importance of PD‐1 in T cell unresponsiveness in other disease models.

## Introduction

Understanding how the immune system is able to deal with a diverse range of pathogens yet remain tolerant to self‐antigens is one of the great challenges in cellular immunology 1. The steady increase in incidence and prevalence of autoimmune diseases across the globe over the past decades emphasises the importance of identifying the mechanisms by which self‐tolerance is maintained 2, 3. Central and peripheral tolerogenic mechanisms play a key role in ensuring that the T cell repertoire is highly diverse yet is sufficiently well regulated to prevent the development of an autopathogenic T cell response 1, 4, 5, 6, 7. Collectively, these mechanisms ensure that the T cell population can respond effectively to a pathogen challenge but that this response does not result in over‐zealous immune activation which would lead to long term damage to the host.

The development of an unresponsive state following exposure to antigen was first termed anergy based on in vitro studies of murine and human T cell clones 8. Anergy can be induced through several approaches, including the presentation of antigen in the absence of co‐stimulation from antigen presenting cells 9, 10 and induction of sustained calcium signalling 11. Key features of anergy include the failure to produce selective cytokines, notably interleukin‐2 (IL‐2), in response to antigen re‐stimulation, the lack of a requirement for antigen to maintain the anergic state and the ability of IL‐2 to reverse anergy 8. In contrast, T cells which develop an unresponsive state post antigen stimulation in vivo appear to have a different phenotype from in vitro anergic cells 12. Typically, T cells which are hyporesponsive to TCR re‐stimulation following primary antigen exposure in vivo down‐regulate the production of all cytokines and require antigen persistence to maintain the unresponsive state, which cannot be reversed by addition of IL‐2. A variety of in vivo manipulations have been applied to induce a hyporesponsive state in T cells which has been termed adaptive tolerance 8, 12, 13, 14, 15, 16, 17, 18, 19.

The regulatory processes or molecules involved in the development and maintenance of an adapted state in T cells in the context of autoantigens are poorly understood. Recently, the co‐inhibitory receptor programmed cell death protein 1 (PD‐1) has been implicated in the development of T cell tolerance following administration of soluble peptide, also termed peptide immunotherapy 20. PD‐1 is upregulated on CD4^+^ T cells following activation and thereby restrains the primary immune response 21. Mice that lack PD‐1 develop spontaneous autoimmune pathology 22 and signalling through PD‐1 after T cell receptor (TCR) stimulation inhibits proliferation of T cells and reduces the production of effector cytokines 23. The discovery of PD‐1/PD‐L1 interaction as a target for checkpoint inhibition has led to several successful clinical trials and the approval of PD‐1 inhibitors as treatment for a number of cancer types 24, 25, 26. However, it remains unclear whether PD‐1 plays a role in the development of T cell adaptation in vivo following exposure to high concentrations of autoantigen under pro‐inflammatory conditions.

In this study we probed the mechanisms and consequences of T cell adaptation in experimental autoimmune encephalomyelitis (EAE), a widely used model of multiple sclerosis. In this model, CD4^+^ T cells are activated by immunisation with CNS antigens in peripheral lymph nodes or ex vivo pre‐activated cells are injected into the bloodstream, and effector T cells consequently make their way into the CNS where they cause inflammation upon re‐encounter with their cognate antigen. We were particularly interested to establish whether adapted T cells could traffic to a site of autoantigen presentation and to define whether increased PD‐1 expression played a critical role in the development of the adapted state.

## Results

### High dose of agonist stimulation in vitro results in CD4^+^ T cell adaptation

**Figure 1:**
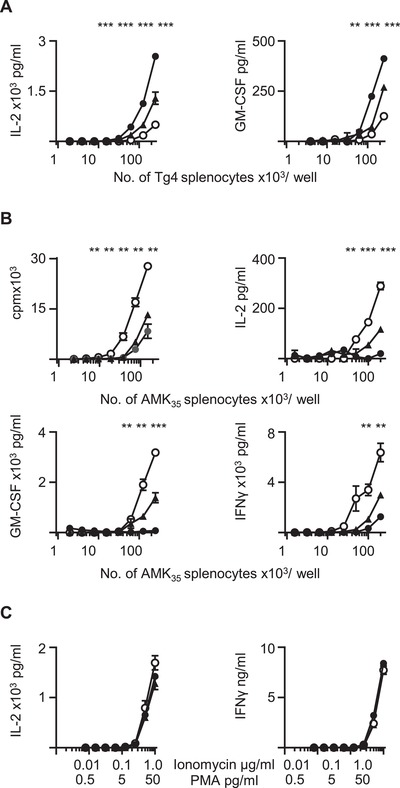
High dose of agonist stimulation in vitro results in CD4^+^ T cell adaptation. (A) Splenocytes isolated from naïve Tg4 transgenic mice were cultured in the presence of 1 μM (dark circle), 0.1 μM (triangle) and 0.01 μM (open circle) 4Tyr MBP, and IL‐2 and GM‐CSF production was analysed by ELISA 48 and 72 h later, respectively. (B) Proliferation (as measured by thymidine incorporation) and IL‐2, IFNγ and GM‐CSF production (analysed by ELISA) of Tg4 TCL cells generated in the presence of 1 μg/mL (dark circle) 0.1 μM (triangle) or 0.01 μM (open circle) of MBP Ac1‐9(4Tyr) following re‐stimulation with varying numbers of splenocytes from AMK_35_ mice expressing MBP. (C) IL‐2 and IFN‐γ production from Tg4 TCL cells generated in the presence of 1 μg/mL (dark circle) 0.1 μM (triangle) or 0.01 μM (open circle) of MBP Ac1‐9(4Tyr), following re‐stimulation with ionomycin and PMA at indicated concentrations. Data are shown as mean±SEM from a single experiment, representative of three independent experiments. Each experiment contained a minimum of three pooled Tg4 spleens (*t*‐test, **p < 0.01, ***p < 0.001).

The Tg4 transgenic mouse provides a source of naïve T cells expressing a TCR with a moderate affinity for the Ac1‐9‐I‐A^u^ complex 27. Substitution of a tyrosine for the native lysine at position 4 increases the MHC binding affinity by approximately 1 million fold, creating a superagonist ligand (4Tyr) for Tg4 cells in vitro 28. Initially, we examined how varying the concentration of MBP Ac1‐9(4Tyr) peptide influenced the proliferation of Tg4 T cells in vitro. As expected, increasing the dose of MBP Ac1‐9(4Tyr) resulted in an increase in proliferation and production of effector cytokines (Fig. 1A). In the next experiment, we examined how the Tg4 T cell lines (TCL) proliferated in a recall assay after initial stimulation with MBP Ac1‐9(4Tyr) peptide. In order to do this we generated Tg4 TCL by initially stimulating Tg4 splenocytes with either 1 μM, 0.1 μM or 0.01 μM MBP Ac1‐9(4Tyr). After 48 h, live cells were isolated and the T cell population was expanded in media enriched with T cell growth factors for a further 4 days prior to isolation of CD4^+^ Tg4 TCL by FACS (Supporting Information Fig. 1). In contrast to the findings during the primary stimulation, we found that Tg4 TCL generated through initial stimulation with 1 μM MBP Ac1‐9(4Tyr) subsequently proliferated less, and produced lower amounts of pro‐inflammatory cytokines, than cells stimulated with initially lower doses of antigen when co‐cultured with irradiated AMK_35_ splenocytes which constitutively express a MBP epitope in MHC class II molecules 29 (Fig. 1B). From this we concluded that initial stimulation with high dose peptide resulted in the generation of a population of hyporesponsive T cells which proliferated less and produced a reduced amount of effector cytokines when stimulated with antigen on a second occasion.

In order to examine whether the adapted state could be overcome through signalling downstream of the TCR, the three Tg4 TCL were stimulated with phorbol myristate acetate (PMA) and ionomycin. As shown in Fig. 1C, all TCL produced similar concentrations of IL‐2 and IFN‐γ in response to PMA and ionomycin. This demonstrated that the adapted state was maintained through differential signalling between the TCR and I‐A^u^‐MBP complex and upstream T cell activation pathways, since re‐stimulation with PMA and ionomycin resulted in equivalent proliferation and effector cytokine production in the three Tg4 TCL.

### Immunisation with high dose of MBP does not result in deletion of MBP‐responsive CD4^+^ T cells

**Figure 2:**
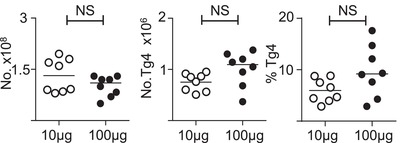
Immunisation with high dose of agonist in‐vivo does not result in deletion of agonist‐responsive CD4^+^ T cells. C57BL/6xB10.PL mice were seeded with CD4^+^CD45.1^+^ Tg4 cells and immunised the following day with either 10 or 100 μg MBP Ac1‐9(4Tyr) and CFA. Mice were sacrificed 6 days following immunisation. Total numbers of splenocytes, as well as numbers and frequencies of CD4^+^CD45.1^+^ Tg4 cells in the spleen at day 6 in mice immunised with either 10 μg (open circles) or 100 μg (dark circles) 4Tyr MBP as assessed by manual counting with a haemocytometer and by flow cytometry. Data are shown as scatter plots with the mean indicated by horizontal bar, from a single experiment representative of two independent experiments with *n* = 6–8 mice per experimental group (Mann–Whitney U test; NS, no significant difference).

The above experiments examined the effects of varying antigen concentration on future T cell phenotypes in vitro. Next, we wanted to examine whether T cells were adapted in vivo following high dose immunisation with MBP Ac1‐9(4Tyr). Host C57BL/6 x B10.PL mice were seeded with Tg4.CD45.1 CD4^+^ T cells and 24 h later were immunised with either 10 μg or 100 μg of MBP Ac1‐9(4Tyr) in Complete Freund's Adjuvant (CFA). Six days later, the mice were sacrificed and FACS analysis was performed on single cell preparations of the spleen (Supporting Information Fig. 2). The total number of cells, number of Tg4 CD4^+^ T cells and the proportion of Tg4 cells in the CD4^+^ population were not significantly different between the two groups of mice (Fig. 2). These observations demonstrate that high dose immunisation of MBP in vivo does not lead to the deletion of MBP responsive CD4^+^ T cells.

### Immunisation with high dose of MBP results in attenuation of EAE

**Figure 3:**
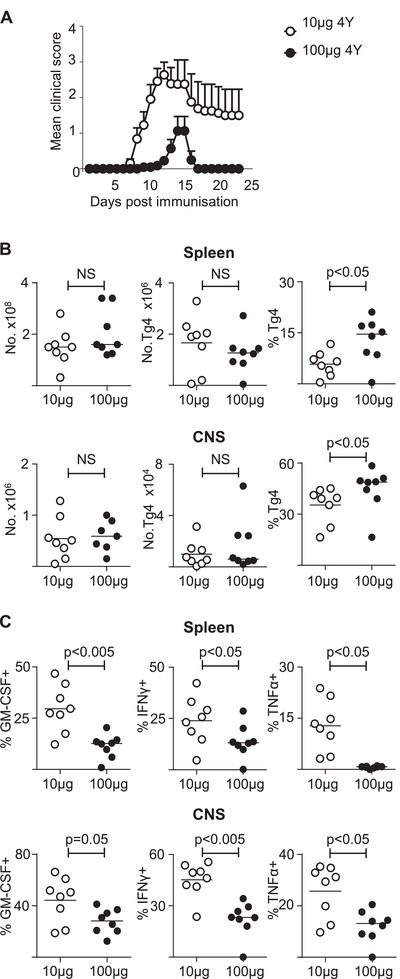
Adapted T cells can access CNS but have significantly reduced pathogenic potential. C57BL/6xB10.PL hosts were seeded with Tg4CD4^+^CD45.1^+^ T cells and 1 day later were immunised with CFA and 10 μg 4Tyr MBP or 100 μg MBP Ac1‐9(4Tyr). (A) The disease course of mice immunised with either 10 μg MBP Ac1‐9(4Tyr) (open circles, *n* = 22) or 100 μg MBP Ac1‐9(4Tyr) (dark circles, *n* = 22) (Fisher's exact test, *p*<0.0005). Differences in peak disease severity between the groups was analysed with Mann–Whitney U test, *p* < 0.0001. Data shown as mean±SEM, pooled from three independent experiments. (B) Mice were sacrificed 12 days following immunisation. Total numbers of cells, as well as numbers of Tg4 cells and frequencies of CD4^+^CD45.1^+^ cells which were Tg4, i.e. donor cells, are shown for the spleen and CNS. (C) Frequencies of splenic or CNS‐infiltrating Tg4 cells producing GM‐CSF, IFN‐γ and TNF‐α following overnight stimulation with 20 μM MBP Ac1‐9(4Lys). Data are shown as scatter plots with the mean indicated by horizontal bar, from a single experiment representative of two independent experiments with *n* = 6–8 per experimental group (Mann–Whitney U test; NS, no significant difference).

In order to examine whether high dose immunisation with MBP could attenuate EAE, we immunised mice with either 10 μg or 100 μg of MBP Ac1‐9(4Tyr) and then monitored the mice daily for motor neurological function. Mice immunised with 10 μg MBP Ac1‐9(4Tyr) developed a synchronous course of EAE whereas mice immunised with 100 μg MBP Ac1‐9(4Tyr) had a significantly lower incidence and severity of EAE (Fig. 3A). Eighteen of 22 mice developed EAE following immunisation with 10 μg MBP Ac1‐9(4Tyr) compared to only 5 of 22 mice immunised with 100 μg MBP Ac1‐9(4Tyr).

To examine whether this was due to a failure of the Tg4 cells to access the CNS following immunisation with 100 μg MBP Ac1‐9(4Tyr), mice in a subsequent experiment were sacrificed 12 days after immunisation. The total number of cells and number of Tg4.CD45.1 CD4^+^ cells were not significantly different in the spleen or CNS between the 2 groups (Fig. 3B). The proportion of Tg4 cells of all CD4^+^ T cells was significantly higher in both the spleen and CNS in mice immunised with 100 μg MBP Ac1‐9(4Tyr) (Fig. 3B, Supporting Information Fig. 3). Following overnight re‐stimulation with 20 μM MBP Ac1‐9(4Lys), the production of cytokines by Tg4 cells was assessed in both groups. In mice immunised with 100 μg MBP Ac1‐9(4Tyr), the proportion of Tg4 cells from both the spleen and CNS expressing pro‐inflammatory cytokines was significantly reduced (Fig. 3C).

### Adapted T cells do not have a regulatory phenotype

**Figure 4:**
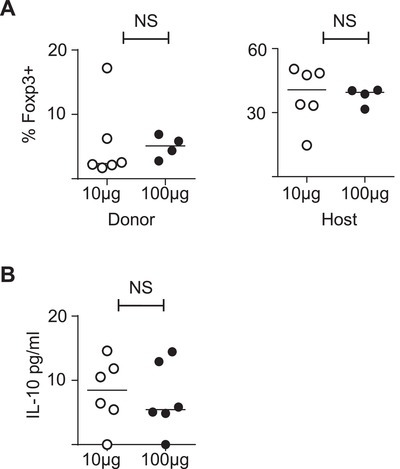
Adapted T cells do not have a regulatory T cell phenotype (A) C57BL/6xB10.PL hosts were seeded with Tg4 CD4^+^CD45.1^+^ T cells and 1 day later immunised with CFA and 10 μg MBP Ac1‐9(4Tyr) or 100 μg MBP Ac1‐9(4Tyr). Mice were sacrificed 12 days following immunisation. Proportions of Fopx3^+^ of CD4^+^CD45.1^+^ (Donor) and CD4^+^CD45.1^−^ (Host) cells in the mononuclear cell preparations from the CNS of mice immunised with 10 μg MBP Ac1‐9(4Tyr) (open circles) or 100 μg MBP Ac1‐9(4Tyr) (dark circles) were determined by flow cytometry. Data are representative of two independent experiments containing 4–6 mice per group (Mann–Whitney U test; NS, no significant difference). (B) Splenocytes of mice 12 days post immunisation with 10 μg MBP Ac1‐9(4Tyr) (open circles) or 100 μg MBP Ac1‐9(4Tyr) (dark circles) were re‐stimulated with 20 μM MBP Ac1‐9(4Lys) overnight and IL‐10 concentrations were measured in the supernatants. Data are shown as scatter plots with the mean indicated by horizontal bar, representative of two independent experiments containing 4–6 mice per experimental group (Mann–Whitney U test; NS, no significant difference).

A potential explanation for the adapted T cell phenotype is that high dose immunisation results in the development of T cells with a regulatory T cell phenotype. In order to examine this possibility, we assessed Foxp3 expression, which is a transcription factor widely used to identify regulatory T cells 7, within Tg4 cells at peak of disease. We found no differences in Foxp3 expression in the transferred Tg4 cells from mice immunised with 10 μg or 100 μg of MBP Ac1‐9(4Tyr) (Fig. 4A). Furthermore, we found no evidence that in vivo adapted cells produced more IL‐10 following overnight re‐stimulation with 20 μM MBP Ac1‐9(4Lys) (Fig. 4B).

### Adapted T cells express higher levels of PD‐1

**Figure 5:**
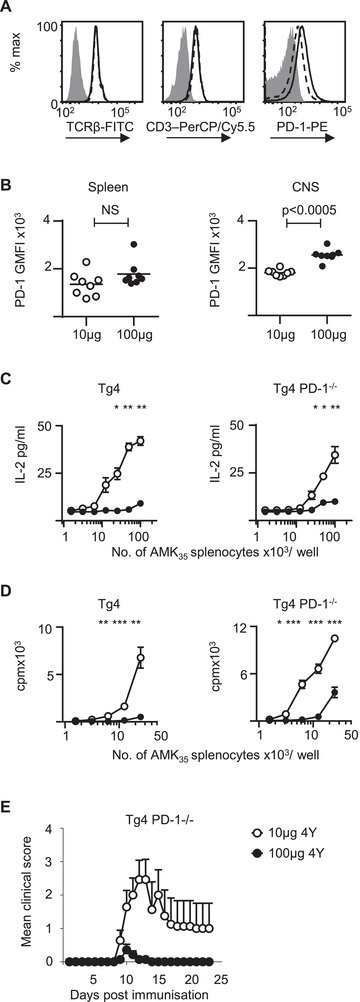
PD‐1 expression is increased on adapted T cells generated in vitro and in vivo but is not required to maintain adapted state (A) Cell surface expression of CD3, TCRβ and PD‐1 and isotype control staining (grey shaded area) on Tg4 TCL cells generated in the presence of 1 μg/mL (solid line) or 0.01 μg/mL (dashed line) of MBP Ac1‐9(4Tyr). Cells were analysed by flow cytometry and gated on CD4^+^ live cells. Data are representative of four independent experiments, with each experiment containing a minimum of 3 pooled Tg4 spleens per experimental group. (B) C57BL/6xB10.PL hosts were seeded with Tg4CD4^+^CD45.1^+^ T cells and 1 day later were immunised with CFA and 10 μg MBP Ac1‐9(4Tyr) (open circle) or 100μg MBP Ac1‐9(4Tyr) (dark circle). Mice were sacrificed 12 days following immunisation. PD‐1 expression on CD4^+^CD45.1^+^ T cells from the spleen and CNS was analysed as mean fluorescence intensity (MFI). Data are shown as scatter plots with the mean indicated by horizontal bar, representative of two independent experiments containing 6–8 mice per experimental group (Mann–Whitney U test; NS, no significant difference). (C) IL‐2 production by Tg4 (left panel) and PD‐1^−/−^ Tg4 (right panel) TCL cells generated in the presence of 1 μg/mL (dark circle) or 0.01 μM (open circle) of MBP Ac1‐9(4Tyr) following re‐stimulation with varying numbers of splenocytes from AMK_35_ mice expressing MBP (*t*‐test, *p < 0.05, **p < 0.01, ***p < 0.001). (D) Proliferation of Tg4 (left panel) and PD‐1^−/−^ Tg4 (right panel) TCL cells generated in the presence of 1 μg/mL (dark circle) or 0.01μM (open circle) of MBP Ac1‐9(4Tyr) following re‐stimulation with varying numbers of splenocytes from AMK_35_ mice expressing MBP, as measured by thymidine incorporation (*t*‐test, *p<0.05, **p < 0.01, ***p < 0.001). (E) C57BL/6xB10.PL mice were seeded with PD‐1^−/−^ Tg4 CD4^+^ T cells and 1 day later immunised with either 10 μg (open circles) or 100 μg (dark circles) MBP Ac1‐9(4Tyr) in CFA and disease course was monitored (Fisher's exact test, *p* < 0.01). Differences in peak disease severity between the groups were analysed with Mann–Whitney U test, *p* < 0.0005. Data are pooled from two independent experiments with a total of 14 mice per experimental group.

In order to probe the mechanism of the adapted state, the cell surface expression of a panel of co‐stimulatory and co‐inhibitory molecules were examined on our three Tg4 TCL by flow cytometry. As shown in Fig. 5A, in vitro adapted T cells did not express altered levels of CD3 or TCRβ but did express higher levels of PD‐1, a molecule which is known to have an important inhibitory effect on T cell activation. The expression of PD‐1 was then examined on Tg4 cells in mice immunised with either 10 μg or 100 μg MBP Ac1‐9(4Tyr). The expression of PD‐1 was significantly increased on Tg4 cells recovered from the CNS, but not the spleen of mice immunised with 100 μg MBP Ac‐1‐9(4Tyr) at the peak of disease (Fig. 5B).

### PD‐1 deficient T cells can still acquire an adapted phenotype in vitro and in vivo

Given the clear association between T cells which had an impaired ability to respond to MBP agonist on re‐stimulation and increased expression of PD‐1, we next wanted to examine whether increased PD‐1 expression was required to maintain the adapted T cell state. T cell lines were generated from Tg4 WT and Tg4.PD‐1^−/−^ 20 splenocytes which were stimulated with either 1, 0.1 or 0.01 μM of MBP Ac1‐9(4Tyr). The TCL generated from both Tg4 WT and Tg4.PD‐1^−/−^ splenocytes initially treated with the high dose of MBP Ac1‐9(4Tyr) displayed the typical adapted phenotype with reduced IL‐2 production (Fig. 5C). A second, independent experiment found the same adapted phenotype in both Tg4 WT and Tg4.PD‐1^−/−^ TCL when using thymidine incorporation rather than IL‐2 as a readout of proliferation (Fig. 5D). To explore whether the loss of PD‐1 altered the ability of Tg4 cells to be adapted in vivo, we seeded host mice with Tg4.PD‐1^−/−^ CD4^+^ cells and immunised the mice with either 10 μg or 100 μg MBP Ac1‐9(4Tyr). Mice immunised with high dose MBP Ac1‐9(4Tyr) developed a significantly attenuated course of EAE which resembled that of mice seeded with Tg4 WT CD4^+^ cells under the same experimental conditions (Fig. 5E, Fig. 3A). These experiments demonstrated that loss of PD‐1 expression did not limit the ability of Tg4 CD4^+^ T cells to be adapted in vitro or in vivo.

## Discussion

The induction of antigen‐specific tolerance is one of the fundamental mechanisms of peripheral tolerance. The utilisation and/or mimicking of in vivo tolerance mechanisms is a promising strategy to achieve antigen‐specific, targeted immunosuppression for immune‐mediated pathologies such as autoimmune diseases, allergy, and allograft rejection 30. Whilst deletion of autoreactive T cells is a known mechanism of peripheral tolerance 31, our study demonstrates that the administration of high dose agonist in conjunction with adjuvant does not invariably lead to wholesale loss of T cells which can respond to this antigen. Instead, we observed an expansion of T cells which had a greatly reduced ability to proliferate and produce pro‐inflammatory cytokines.

Tolerance against autoantigens is most commonly observed upon administration or presentation in the absence of pro‐inflammatory stimuli. However, high doses of antigen or chronic exposure to antigen, best studied in the context of viral infection and cancer, can result in a hyporesponsive T cell state even in the presence of immune stimuli that support immune responses at other levels of antigen exposure. In the context of autoimmune disease, molecular mimicry is one of the hypothesised mechanisms by which autopathogenic immune responses can be initiated 6. It involves an initial peripheral infection to activate T cells which also recognise self‐antigen due to TCR degeneracy; these then go on to cause an autoimmune response upon encounter of this self‐antigen if not tolerised. One way of achieving tolerance in this setting would be a reduction of T cell trafficking to the site of self‐antigen, in the case of this experimental set‐up, the CNS. However, we found that the hyporesponsive, adapted T cells could still traffic to the CNS. Indeed, the Tg4 CD4^+^ T cells capable of responding to MBP were present in similar numbers in the CNS of asymptomatic mice immunised with high dose MBP as mice with EAE which had been administered a lower dose of MBP. In both groups, there were significant numbers of mononuclear cells in the CNS which were not Tg4 cells. Whilst the Tg4 CD4^+^ T cells are essential for initiating disease in B10.PL × C57BL/6 mice immunised with low dose MBP 32, non Tg4 immune cells are likely to play a role in propagating CNS pathology. This indicates that the CNS was exposed to a similar number of MBP responsive cells but the process of adaptation had rendered them unable to initiate autoimmune pathology. This process may be important in protecting the host from the development of an autopathogenic immune response when the immune system is exposed to autoantigens in an inflammatory milieu through trauma or infection. Epidemiological data supports this hypothesis since a traumatic event does not invariably precede the development of autoimmunity 33. Whether our observations can be readily extrapolated to other antigens beyond modified MBP and ultimately, to polyclonal T cell responses, remains to be clarified.

In recent years, it has been established that PD‐1 is an important molecule in restraining T cell effector function following initial activation. The first evidence for the role of PD‐1 in modulating T cell activation came from PD‐1 deficient mice which developed multi‐organ autoimmunity 34. Interaction of PD‐1 with its ligand PD‐L1 or PD‐L2 and downstream signalling results in the attenuation of the functional and proliferative capabilities of T cells, e.g. via repression of TCR signalling 35, induction of inhibitory genes 36, and reduction of T cell motility 37. The therapeutic blockade of PD‐1 has been a major breakthrough in the treatment of several types of cancer, facilitating a more effective cytotoxic T cell response 22, 25, 38. On the other hand, enhancement of the PD‐1/PDL‐1 axis is considered a promising target in the context of autoimmune disease. A recent study from our group demonstrated that the effectiveness of peptide immunotherapy is reliant on persistent expression of PD‐1 on tolerised T cells 20. Data from the current study show that upregulated expression of PD‐1 is maintained on hyporesponsive T cells which have been generated either in vitro or in vivo. However, using T cells which were deficient in PD‐1, we were able to demonstrate that PD‐1 expression is not required to develop a hyporesponsive state when high levels of autoantigen agonists are delivered alongside a powerful pro‐inflammatory adjuvant. Why PD‐1 expression is indispensable for the maintenance of a hyporesponsive state in Tg4 CD4^+^ cells tolerised by soluble peptide, but not in hyporesponsive T cells generated post peptide administration with CFA, remains unclear but may relate to the induction of an alternative inhibitory pathway driven by the adjuvant 39. A better understanding of the distinct hyporesponsive T cell states, their development, maintenance, and relevance in immune homeostasis and disease is required in order to optimise therapeutic approaches targeting T cell responsiveness 40.

A hallmark of regulatory T cells is unresponsiveness to antigen stimulation, so a plausible explanation for the persistence of hyporesponsive cells following high dose immunisation with MBP is the development of a regulatory T cell phenotype. However, we found no evidence that adapted T cells – although unresponsive to secondary antigen stimulation – acquired the expression of transcription factor Foxp3, a marker for thymus‐derived as well as peripherally‐induced regulatory T cells 7, 41, 42. Likewise, no increase in production of IL‐10, a potent anti‐inflammatory cytokine produced by Foxp3^+^ as well as Foxp3^−^ T cells with regulatory function was observed in adapted T cells 7. A limitation of our experimental approach was the failure to assess IL‐10 production from Tg4 using intracellular cytokine approach as we employed in the assessment of pro‐inflammatory cytokine production. The finding that adapted T cells in our experimental system did not acquire a regulatory T cell phenotype is consistent with other studies which have investigated the in vivo development of a hyporesponsive state in T cells post antigen exposure 43.

In conclusion, we show that a high dose of self‐antigen agonist in the presence of adjuvant results in T cell tolerance, which follows initial T cell expansion and does not involve clonal deletion, autoantigen ignorance, or acquisition of a regulatory phenotype. Most strikingly, this in vivo adapted T cell phenotype is independent of PD‐1, which stands in contrast with several other described forms of T cell unresponsiveness, including in the context of chronic viral infections and cancer. These insights into adapted T cell tolerance are important in the development of antigen‐specific therapy for autoimmune diseases.

## Materials and methods

### Mice, antigens and tissue culture medium

B10.PL x C56BL/6, AMK_35_,Tg4.CD45.1 and Tg4.CD90.1 mice were bred under specific pathogen‐free conditions at the University of Edinburgh. All experiments had local ethical approval and were performed in accordance with UK legislation. Tg4 mice express a transgenic T cell receptor (TCR) recognizing the Ac1‐9 peptide of MBP in association with I‐A^u^ 27. AMK_35_ mice express the MBP Ac1‐9 peptide covalently bound to all I‐A^U^ major histocompatibility molecule (MHC) class II molecules 29. The MBP Ac1‐9(4Tyr) analog peptide was obtained from Cambridge Research Biochemicals (Cleveland, UK). Tissue culture medium (RPMI 1640 medium) was supplemented with 2 mM L‐glutamine, 100 U/ml penicillin, 100 μg/ml streptomycin, and 5 × 10^–5^ M 2‐ME (all from Invitrogen Life Technologies, Paisley, UK) and 10% FCS (Sigma‐Aldrich, Dorset, UK).

### Induction of active EAE

B10.PL x C57BL/6 (CD45.2) mice received 1 × 10^6^ Tg4.CD45.1 CD4^+^ T cells. One day later (day 0), mice received 10 μg or 100 μg of the MBP Ac1‐9(4Tyr) peptide emulsified in CFA containing 50 μg of heat‐killed *Mycobacterium tuberculosis* H37Ra (Sigma‐Aldrich, Dorset, UK) at a total final volume of 50 μl injected s.c. into each hind leg. On the day of immunization and 48 hrs later, each mouse also received 200 ng of pertussis toxin (Health Protection Agency, Dorset, U.K.) in 0.5 mL PBS via i.p. injection. Clinical signs of EAE were assessed daily with the following scoring system: 0, no signs; 1, flaccid tail; 2, impaired righting reflex and/or gait; 3, partial hind limb paralysis; 4, total hind limb paralysis; 5, hind limb paralysis with partial front limb paralysis; 6, moribund or dead. In some experiments, the mice were sacrificed pre disease development on day 6 or at peak of disease at day 12 post immunisation.

### Generation of Tg4 T cell lines, primary activation assays and recall assays

To study the primary activation of Tg4 CD4^+^ T cells, spleens from Tg4 mice were harvested, single cell preparation was performed and red blood cells were lysed using an ammonium chloride buffer (Sigma Aldrich, Dorset, UK). Varying numbers (as stated) of Tg4 splenocytes cells per well (flat bottomed, 96 well plates) were cultured with stated concentration of MBP Ac1‐9(4Tyr). After 48 h, cell proliferation was assessed by the addition of [^3^H]thymidine (PerkinElmer, Cambridge, UK) at 0.5μCi/well for the last 18 h of culture. [^3^H]Thymidine incorporation was measured using a scintillation β‐counter (Wallac, Milton Keynes, UK). The results are expressed as mean counts per minute (c.p.m.) ± standard error of the mean (SEM). Tg4 CD4^+^ T cell production of cytokines (IL‐2 and IFN‐γ) was assessed in culture supernatants by ELISA using paired monoclonal antibodies and recombinant cytokine standards purchased from BD Biosciences (New Jersey, USA). GM‐CSF was detected using Ready‐SET‐Go ELISA kits (eBioscience, San Diego, USA). IL‐2 was measured in supernatants after 48 h of culture and IFN‐γ and GM‐CSF was measured after 72 h of culture.

Tg4 CD4^+^ T cell lines were generated by culturing Tg4 splenocytes with the stated concentration of MBP Ac1‐9(4Tyr) for 48 h. Cells were plated out in 24‐well plates at 3 × 10^6^/mL with 1.5 mL in each well. T cell blasts were then isolated using Ficoll‐Paque 1.077 (GE Healthcare Life Sciences, Buckinghamshire, UK). The isolated cells were expanded for a further 4 days at 2 × 10^6^/mL in tissue culture media supplemented with 2.5% concanavalin A‐activated rat spleen supernatant as a source of T cell growth factors in 24 well plates. To examine response to recall stimulation to MBP, 2 × 10^4^/ well Tg4 CD4^+^ TCL were co‐cultured in 96 well plates with the stated concentration of irradiated (30 Gy) AMK_35_ splenocytes for 72 h. Response to a non TCR activation stimulus was examined by culturing 1 × 10^5^/ well Tg4 TCL with the stated amount of ionomycin and PMA. Proliferation and cytokine production were assessed as described above.

### Flow cytometry analysis

Cells were re‐suspended in FACS buffer (PBS, 2% fetal calf serum, 0.01% sodium azide) (Sigma Aldrich, Dorset, U.K.). Mice with EAE were sacrificed by CO_2_ asphyxiation, perfused with cold PBS and mononuclear cells were prepared from the brain and spinal cord as described previously 44. Spinal cords were removed by intrathecal hydrostatic pressure. Brain and spinal cords were cut into small pieces and digested with 2.5 mg/mL collagenase (Worthington Biochemical) and 1 mg/mL deoxyribonuclease (Sigma‐Aldrich) for 25minutes at 37°C followed by mechanical disaggregation. Single cell suspensions were washed once in tissue culture medium. Mononuclear cells from the CNS were prepared from the interface of a 30:70% discontinuous Percoll gradient after centrifugation for 20 minutes at 850 × g. Cells were re‐suspended in 1mL tissue culture medium and cell numbers were manually determined using a cell counting chamber, with the addition of 0.4% Trypan Blue for exclusion of dead cells. Analysis of CNS mononuclear phenotype and function was performed by flow cytometry as described in relevant methods and results sections, and adhered to guidelines as described in Cossarizza *et al*. 45. The number of Tg4 donor cells was calculated based on the percentage of CD45.1^+^ cells of live singlet cells as determined by flow cytometric analysis. Fc receptors were blocked with supernatant from the hybridoma 2.4G2. All antibodies used were from eBioscience, Hatfield, UK, except where stated; LIVE/DEAD fixable cell stain (Life Technologies), anti‐CD4‐PerCP, anti‐CD4‐AF700 (BD Pharmingen, Oxford, UK), anti‐CD90.1‐APC, anti‐CD45.1‐FITC, anti‐TNFα‐efluor450, anti‐IFNγ‐FITC, anti‐GM‐CSF‐PE and anti‐Foxp3‐PE (AbD Serotec, Kidlington, UK). For intracellular staining in response to peptide, cells were re‐suspended at 1 × 10^7^/mL in the presence or absence of 20 μM 4Lys MBP. After overnight culture, 1 μl/mL of brefeldin A (e‐bioscience, Hatfield, UK, 1000x stock) was added for the last four hours of culture. Cells were surface stained prior to processing for intracellular staining using appropriate buffers according to manufacturer's instructions (eBioscience for transcription factor staining or Becton Dickinson for cytokine staining). FACS data were collected using LSR Fortessa (BD Biosciences, New Jersey, USA) and analyzed using FlowJo software (Tree Star, Olten, Switzerland). For FACS analysis of CNS mononuclear cells, mice with EAE were sacrificed by CO_2_ asphyxiation, perfused with cold PBS and mononuclear cells were prepared from brain and spinal cord as described previously 44. IL‐10 was measured in supernatants of splenocytes stimulated overnight with MBP using a IL‐10 Ready‐SET‐Go ELISA (eBioscience, San Diego, USA).

### Statistical analysis

Statistical analysis of results was performed using the Mann–Whitney U test, the two tailed Student's *t‐*test and Fischer's exact test as appropriate. Cytokine concentrations are presented as mean concentration ± SEM. Significance was set at *p* < 0.05.

## Conflict of interest

The authors declare no commercial or financial conflict of interest.

**Abbreviations**
CFA: Complete Freund's Adjuvant
CNS: central nervous system
EAE: experimental autoimmune encephalomyelitis
IL‐: interleukin
Lys: lysine
MBP: myelin basic protein
PD‐1: programmed cell death protein 1
PMA: phorbol myristate acetate
TCL: T cell line
TCR: T cell receptor
Tyr: tyrosine

## Supporting information

Peer review correspondence

Supporting Information figure 1: Demonstration of gating strategy for the identification of CD4^+^ Tg4 TCL. Following in vitro culture of Tg4 splenocytes to obtain a Tg4 TCL, CD4^+^ T cells were identified by successively gating for cells, singlets, live cells, and CD4^+^ cells using appropriate markers.
Supporting Information figure 2: Demonstration of gating strategy for the identification of CD4^+^CD45.1^+^ Tg4 donor cells in the spleen of host CD45.2 mice at day 6 post immunisation. CD4^+^ Tg4 donor cells were distinguished from host CD4^+^ T cells by surface expression of CD45.1.
Supporting Information figure 3: (A) Demonstration of gating strategy for the identification of donor CD4^+^CD45.1^+^ Tg4 T cells in the CNS of host CD45.2 mice at day 12 post immunisation. CD4^+^ Tg4 donor cells were distinguished from host CD4^+^ T cells by surface expression of CD45.1. (B) Demonstration of gating strategy for identification of intracellular cytokine production following overnight stimulation with MBP.
