# Supplementary material for: PD‐1 expression is upregulated on adapted T cells in experimental autoimmune encephalomyelitis but is not required to maintain a hyporesponsive state
Source: Eur J Immunol. 2018 Dec 7;49(1):112–20. doi: 10.1002/eji.201847868 (PMC6492152; doi:10.1002/eji.201847868)
Supplement: Supplementary file 2 — Supporting Information figure 1: Demonstration of gating strategy for the identification of CD4+ Tg4 TCL. Following in vitro culture of Tg4 splenocytes to obtain a Tg4 TCL, CD4+ T cells were identified by successively gating for cells, singlets, live cells, and CD4+ cells using appropriate markers. Supporting Information figure 2: Demonstration of gating strategy for the identification of CD4+CD45.1+ Tg4 donor cells in the spleen of host CD45.2 mice at day 6 post immunisation. CD4+ Tg4 donor cells were distinguished from host CD4+ T cells by surface expression of CD45.1. Supporting Information figure 3: (A) Demonstration of gating strategy for the identification of donor CD4+CD45.1+ Tg4 T cells in the CNS of host CD45.2 mice at day 12 post immunisation. CD4+ Tg4 donor cells were distinguished from host CD4+ T cells by surface expression of CD45.1. (B) Demonstration of gating strategy for identification of intracellular cytokine production following overnight stimulation with MBP. [file EJI-49-112-s002.pdf]

European Journal of Immunology

Supporting Information
for
DOI 10.1002/eji.201847868

Iris Mair, Dario Besusso, Louise Saul, Sarju D. Patel, Rahul Ravindran, Rhoanne C. McPherson, Melanie D. Leech, Richard A. O'Connor, Stephen M. Anderton and Richard J. Mellanby

PD-1 expression is upregulated on adapted T cells in experimental autoimmune encephalomyelitis but is not required to maintain a hyporesponsive state

© 2018 The Authors. *European Journal of Immunology* published by WILEY-VCH Verlag GmbH & Co. KGaA, Weinheim.

Supplementary Figure 1

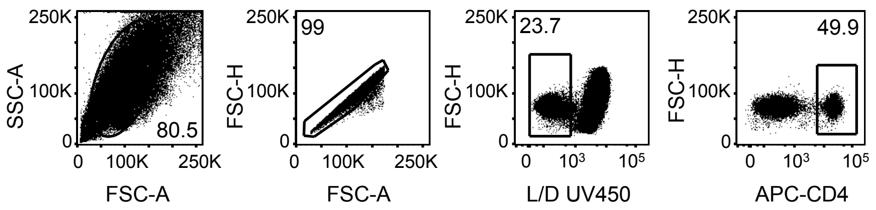

Supplementary figure 1: Demonstration of gating strategy for the identification of $CD4^+$ Tg4 TCL. Following *in vitro* culture of Tg4 splenocytes to obtain a Tg4 TCL, CD4+ T cells were identified by successively gating for cells, singlets, live cells, and CD4+ cells using appropriate markers.

Supplementary Figure 2

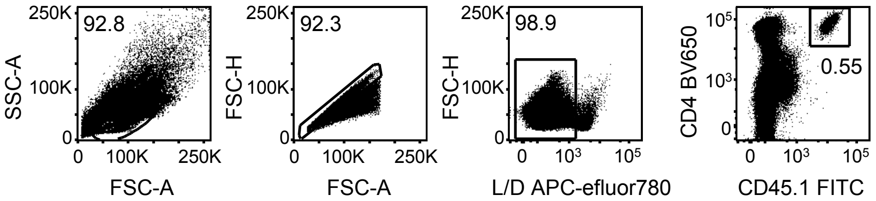

Supplementary figure 2: Demonstration of gating strategy for the identification of CD4$^+$CD45.1$^+$ Tg4 donor cells in the spleen of host CD45.2 mice at day 6 post immunisation. CD4$^+$ Tg4 donor cells were distinguished from host CD4$^+$ T cells by surface expression of CD45.1.

Supplementary Figure 3

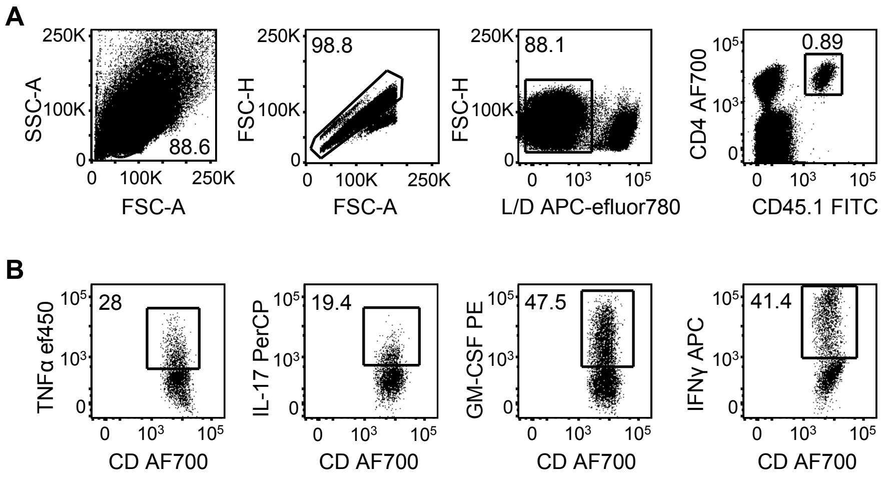

Supplementary figure 3: (A) Demonstration of gating strategy for the identification of donor CD4⁺CD45.1⁺ Tg4 T cells in the CNS of host CD45.2 mice at day 12 post immunisation. CD4⁺ Tg4 donor cells were distinguished from host CD4⁺ T cells by surface expression of CD45.1. (B) Demonstration of gating strategy for identification of intracellular cytokine production following overnight stimulation with MBP.
