# Supplementary material for: PD‐1 expression is upregulated on adapted T cells in experimental autoimmune encephalomyelitis but is not required to maintain a hyporesponsive state
Source: Eur J Immunol. 2018 Dec 7;49(1):112–20. doi: 10.1002/eji.201847868 (PMC6492152; doi:10.1002/eji.201847868)
Supplement: Supplementary file 1 — Peer review correspondence [file EJI-49-112-s001.pdf]

European Journal of Immunology

PD-1 expression is upregulated on adapted T cells in experimental autoimmune encephalomyelitis but is not required to maintain a hyporesponsive state

Mair, Iris; Besusso, Dario; Saul, Louise; Patel, Sarju; Ravindran, Rahul; McPherson, Rhoanne; Leech, Mel; O'Connor, Richard; Anderton, Stephen; Mellanby, Richard

Correspondence: Prof. Richard Mellanby

University of Edinburgh

Edinburgh

EH25 9RG

Review Timeline:	Submission date:	16-Aug-2018
	First Editorial decision:	19-Sep-2018
	Revision/s received:	09-Oct-2018; 24-Oct-2018
	Second Editorial decision:	18-Oct-2018
	Final Revision received:	24-Oct-2018
	Accepted:	09-Nov-2018

Handling Executive Committee member: Prof. Britta Engelhardt

Please note that the correspondence below does not include the standard editorial instructions regarding preparation and submission of revised manuscripts, only the scientific revisions requested and addressed.

First Editorial Decision

19-Sep-2018

Dear Prof. Mellanby,

Manuscript ID eji.201847868 entitled "PD-1 expression is upregulated on adapted T cells but is not required to maintain a hyporesponsive state" which you submitted to the European Journal of Immunology has been reviewed. The comments of the referees are included at the bottom of this letter. Please accept our apologies for a delay in the peer review of your manuscript.

Please note that referees have raised a number of issues that may require longer time than the revision

timeframe of 6 months. Please let us know if you think the suggested experiments will be feasible for you finish within the mentioned timeframe. A revised version of your manuscript that takes into account the comments of the referees will be reconsidered for publication. Should you disagree with any of the referees' concerns, you should address this in your point-by-point response and provide solid scientific reasons for why you will not make the requested changes.

You should also pay close attention to the editorial comments included below. **In particular, please edit your figure legends to follow Journal standards as outlined in the editorial comments. Please provide full gating strategy and fluorochrome axis labels for all flow cytometry plots. Failure to do this will result in delays in the re-review process.**

Please note that submitting a revision of your manuscript does not guarantee eventual acceptance, and that your revision will be re-reviewed by the referees before a decision is rendered.

If the revision of the paper is expected to take more than three months, please inform the editorial office. Revisions taking longer than six months may be assessed by new referees to ensure the relevance and timeliness of the data.

Once again, thank you for submitting your manuscript to European Journal of Immunology and we look forward to receiving your revision.

Yours sincerely,
Ivan Shevchenko

On behalf of
Prof. Britta Engelhardt

Dr. Ivan Shevchenko
Editorial Office
European Journal of Immunology
e-mail: ejied@wiley.com
www.eji-journal.eu

European Journal of Immunology

****

Reviewer: 1

Comments to the Author

The current study addresses the role of antigen dose and PD-1 in adaptive tolerance. The authors show that antigen dose is an important parameter in induction of adaptive tolerance, and prevention of CNS autoimmunity, but not CNS infiltration. Moreover, the authors show that PD-1 is not responsible for tolerance induction. This is a clear and well-written study; however, certain points could benefit from additional analysis or clarification. Specific comments follow.

Minor comments:

In the disease induction model the endogenous polyclonal T cells could potentially be recruited to the response in either tolerogenic or pro-inflammatory role. For example, does Foxp3% change in non-TCR transgenic T cells after immunization with high or low dose peptide? Can polyclonal endogenous T cells contribute to disease development at the low dose immunization? In Figure 3 it seems that at least 50% of cells infiltrating the CNS are not TCR transgenic.

The Y-axis in Figure 3B panels for %Tg4 could use additional tick marks.

Since the self-peptide used in the study is modified, it might be more appropriate to refer to it as agonist or strong agonist, rather than autoantigen (For example Discussion line 248). Similarly, "MBP" nomenclature suggests unmodified protein rather than modified peptide. It is possible that unmodified autoantigen would not have the same effect. The difference between immunizing peptide and self-antigen presented in the CNS is an important distinction.

In addition to antigen dose/antigen availability, peptide:MHC complex stability, TCR affinity, and the context of peptide presentation (inflammatory CFA adjuvant vs. tolerogenic peptide) could affect the type and degree of tolerance induction. Since the study utilizes a single TCR with single TCR affinity for antigen, it is unknown whether polyclonal responses would be similarly affected.

Statistical analysis is missing from Figures 3A and 5D.

Reviewer: 2

Comments to the Author

The study by Mair et al presents a clearly outlined and nicely presented investigation into the generation and maintenance of hyporesponsive T cells. The work is logically presented and presents both in vitro and in vivo evidence that stimulation with high levels of antigen can lead to a hyporesponsive state that can be rescued by activation of downstream signalling pathways. This hyporespontsive state in vivo leads to a reduced incidence and severity of EAE and reduced T cell cytokine production in the spleen and CNS. The authors conclude that Tregs are not altered and that while PD-1 is unregulated it is not essential for the hyporesponsive state or reduction in EAE. While nicely written and presented, the authors make several claims that are not supported by the data presented. Additionally, the experimental detail and methods are not well described or justified in some cases.

Specific points:

1. The introduction is well written but is too long to introduce this work. Much of the precise details are reiterated in the Discussion and so this section could be considerably shortened.
2. One issue that is not considered when comparing the high versus low dose stimulation of T cells is the kinetics of the response. Does the high Ag dose simply drive the T cells to a hyporesponsive state sooner than the lower doses? If so, would assessing responses at an earlier or later time point alter the conclusions?
3. The results shown in Figure 2b are from one single experiment with pooled spleens. Thus, what is shown are technical replicates and does not necessarily show the biological variability of the system. Statistics are not useful for this single experiment. The authors need to show the combined results from several independent experiments to be able to claim that the differences they have observed are not due to chance.
4. Figure 3b shows the number of cells in the CNS. How have these numbers been calculated? The authors need to show their flow cytometric gating strategy to enable the reader to understand what the data presented mean. It is also clear that the Tg4 T cells comprise only a small percentage of the total number of cells; what are the other cells in the CNS? This question becomes important as the authors

show that there is an increase in the % of CD4 T cells that are Tg4 cells in the CNS. Given that the total number of cells has not changed and the total number of Tg4 cells has not changed, the only explanation is that the number of non-Tg4 CD4 T cells has decreased. If so, how does this change affect the authors' conclusions? In general, the authors pointedly ignore all of the non-Tg4 cells in the spleen and CNS. Given the possible contribution of these cells to the in vivo disease model, this part of the study needs to be more carefully considered and more fully presented.

5. One of the key findings in this manuscript is that Tregs are not altered; however, the data do not support this conclusion. Figure 4a shows the % of FoxP3+ cells in the spleen but % of what? What about the absolute numbers? Additionally, as shown in Figure 5, it is the presence of Tregs in the CNS that is key. These data need to be included. Finally, IL-10 production is assessed in supernatants from splenocytes, but to be consistent with the other data presented, the authors should show intracellular IL-10 by Tg4 cells in the CNS and spleen. As it stands, the authors need to either provide conclusive evidence that Tregs are not altered or remove this section.

6. Figure 5c like Figure 2b shows data from one experiment using pooled cells. To draw any conclusions regarding the biology, the authors need to present the combined data from all experiments.

First Revision – authors' response

Dear Editor,

We would like to thank you for providing the constructive and informative feedback on our manuscript. The comments have undoubtedly improved the quality of the paper and we have addressed each concern in italics below.

If you require any further information please do not hesitate to contact me,

Yours sincerely,

Richard Mellanby

Reviewer: 1

Comments to the Author

The current study addresses the role of antigen dose and PD-1 in adaptive tolerance. The authors show that antigen dose is an important parameter in induction of adaptive tolerance, and prevention of CNS autoimmunity, but not CNS infiltration. Moreover, the authors show that PD-1 is not responsible for tolerance induction. This is a clear and well-written study; however, certain points could benefit from additional analysis or clarification. Specific comments follow.

Minor comments:

In the disease induction model the endogenous polyclonal T cells could potentially be recruited to the response in either tolerogenic or pro-inflammatory role. For example, does Foxp3% change in non-TCR transgenic T cells after immunization with high or low dose peptide? Can polyclonal endogenous T cells contribute to disease development at the low dose immunization? In Figure 3 it seems that at least 50% of cells infiltrating the CNS are not TCR transgenic.

We thank the reviewer for making this point. In the non-transgenic CD4 T cells, there is no significant difference in the proportion of CD4+ T cell which express foxp3. This information has now been added in to Figure 4. The central drivers of the Tg4-B10.PLxC57BL/6 active EAE model are the transferred T cells. Our initial studies in this model described that immunization with CFA/MBP only drives disease if the host B10.PLxC57BL/6 mice are pre-seeded with MBP responsive Tg4 cells (1). Therefore, whilst invariably other cells infiltrate the inflamed CNS, the key cells which initiate the pathogenic process are the MBP responsive T cells.

The Y-axis in Figure 3B panels for %Tg4 could use additional tick marks.

We have altered the Y axis in the above panels as suggested.

Since the self-peptide used in the study is modified, it might be more appropriate to refer to it as agonist or strong agonist, rather than autoantigen (For example Discussion line 248). Similarly, “MBP”

nomenclature suggests unmodified protein rather than modified peptide. It is possible that unmodified autoantigen would not have the same effect. The difference between immunizing peptide and self-antigen presented in the CNS is an important distinction.

We thank the reviewer for highlighting this point. We have changed autoantigen to agonist as suggested in the result section and figure legends.

In addition to antigen dose/antigen availability, peptide:MHC complex stability, TCR affinity, and the context of peptide presentation (inflammatory CFA adjuvant vs. tolerogenic peptide) could affect the type and degree of tolerance induction. Since the study utilizes a single TCR with single TCR affinity for antigen, it is unknown whether polyclonal responses would be similarly affected.

We acknowledge that our study was only focussed on the MBP-Tg4 system and that our conclusions cannot be immediately transferred to other experimental systems involving polyclonal responses. This point has been highlighted in the discussions (lines 274-276).

Statistical analysis is missing from Figures 3A and 5D.

The statistical results, including the name of the statistical test employed, have now been added to figure legends 3A and 5D.

Reviewer: 2

Comments to the Author
The study by Mair et al presents a clearly outlined and nicely presented investigation into the generation and maintenance of hyporesponsive T cells. The work is logically presented and presents both in vitro and in vivo evidence that stimulation with high levels of antigen can lead to a hyporesponsive state that can be rescued by activation of downstream signalling pathways. This hyporespontsive state in vivo leads to a reduced incidence and severity of EAE and reduced T cell cytokine production in the spleen and CNS. The authors conclude that Tregs are not altered and that while PD-1 is unregulated it is not essential for the hyporesponsive state or reduction in EAE. While nicely written and presented, the authors make several claims that are not supported by the data presented. Additionally, the experimental detail and

methods are not well described or justified in some cases.

We thank the reviewer for reporting that our work is clearly outlined, nicely presented and logical. We hope that our responses below ensure that claims are now fully supported. We also hope that the experimental detail and methods are now well described and justified throughout.

Specific points:

1. The introduction is well written but is too long to introduce this work. Much of the precise details are reiterated in the Discussion and so this section could be considerably shortened.

We have shortened the introduction as suggested.

2. One issue that is not considered when comparing the high versus low dose stimulation of T cells is the kinetics of the response. Does the high Ag dose simply drive the T cells to a hyporesponsive state sooner than the lower doses? If so, would assessing responses at an earlier or later time point alter the conclusions?

We agree that examining different timepoints is important to understand the kinetics of the onset and stability of T cell adaptation, which is why we took the approach of examining the phenotype and function of hyporesponsive T cells generated following in vivo immunization with high dose Ac1-9 4Y MBP at day 6 (at the height of the immune response in draining lymph nodes), day 12 (onset of disease in mice immunized with low dose antigen), as well as after 23 days (past peak of disease in mice immunized with low dose antigen, figures 3A and 5D).. Indeed, in some of these experiments, the model was allowed to run for several additional days and no late onset disease was observed in any of the mice immunized with high dose Ac1-9 4Y MBP.

3. The results shown in Figure 2b are from one single experiment with pooled spleens. Thus, what is shown are technical replicates and does not necessarily show the biological variability of the system. Statistics are not useful for this single experiment. The authors need to show the combined results from several independent experiments to be able to claim that the differences they have observed are not due to chance.

It is challenging to mimic the in-vitro experiments described in Figure 1 given that we recovered insufficient cells from each mouse to allow those experiments to be repeated on an individual mouse basis; therefore, we had no option but to pool the cells from several mice in order to examine the proliferative and cytokine response at multiple antigen concentrations. We acknowledge that pooling data from experiments can be useful and have taken this approach in the disease course study described in figures 3A and 5D. This is made feasible by the binary disease/ no disease statistical analysis and the limited variation in scores each mouse can be awarded (ie 0-4). In recall assays, the absolute amount of proliferation and cytokines produced inevitably varies between experiments although the differential response between Tg4 cells from mice immunized with either 100µg or 10µg is reproduced. This prevents us from meaningfully combining experiments and why, as we have done with ELISA results from TCL, we have elected to present representative data from a single experiment. If this approach remains unacceptable to the reviewer after our explanation, we are happy to remove the figure since it is not critical to our manuscript.

4. Figure 3b shows the number of cells in the CNS. How have these numbers been calculated? The authors need to show their flow cytometric gating strategy to enable the reader to understand what the data presented mean. It is also clear that the Tg4 T cells comprise only a small percentage of the total number of cells; what are the other cells in the CNS? This question becomes important as the authors show that there is an increase in the % of CD4 T cells that are Tg4 cells in the CNS. Given that the total number of cells has not changed and the total number of Tg4 cells has not changed, the only explanation is that the number of non-Tg4 CD4 T cells has decreased. If so, how does this change affect the authors' conclusions? In general, the authors pointedly ignore all of the non-Tg4 cells in the spleen and CNS. Given the possible contribution of these cells to the in vivo disease model, this part of the study needs to be more carefully considered and more fully presented.

The number of cells in the CNS were calculated by counting the number of cells in the CNS preparations. We apologize for not including this information in the methods of the initial draft and this has now been corrected in the revised text (lines 382-384). A wide range of other cells are present in the immune cell infiltrates including CD11b+ cells. The gating strategy for this analysis is now shown in the supplementary figures.

Whist we acknowledge that other cells are present in the CNS at peak of disease, it is also important to

acknowledge that the central drivers of the Tg4-B10.PLxC57BL/6 active EAE model are the transferred T cells. Our initial studies in this model described that immunization with CFA/MBP only drives disease if the host B10.PLxC57BL/6 mice are pre-seeded with MBP responsive Tg4 cells; without the T cell transfer, immunization of B10.PLxC57BL/6 mice with MBP does not induce disease (1). Therefore, whilst invariably other cells infiltrate the inflamed CNS, the key cells which initiate the pathogenic process are undoubtedly the MBP responsive T cells.

5. One of the key findings in this manuscript is that Tregs are not altered; however, the data do not support this conclusion. Figure 4a shows the % of FoxP3+ cells in the spleen but % of what? What about the absolute numbers? Additionally, as shown in Figure 5, it is the presence of Tregs in the CNS that is key. These data need to be included. Finally, IL-10 production is assessed in supernatants from splenocytes, but to be consistent with the other data presented, the authors should show intracellular IL-10 by Tg4 cells in the CNS and spleen. As it stands, the authors need to either provide conclusive evidence that Tregs are not altered or remove this section.

We apologize for the lack of clarity in this figure which we have now corrected in the figure legend. The Foxp3+ data is from CNS T cells and so we consider our interpretation that there is no change in donor Foxp3+ T cells in the CNS to be robust. We have now also added a complementary figure on the proportion of Foxp3+ T cells in the host population. The proportion of Foxp3+ cells in the Tg4 population remains low in both experimental groups compared to the host CD4+ population. Furthermore, we did not find any significant difference in the absolute numbers of the Foxp3+ cells in the CNS of mice immunized with either 10µg or 100µg 4Y Ac1-9 MBP.

To further investigate the possibility that the adapted T cells have a regulatory phenotype, we examined the absolute numbers and proportion of Foxp3+ cells in the donor Tg4 population at the day 6 timepoint post immunization with either 10µg or 100µg 4Y Ac1-9 MBP (i.e. in the experiment reported in figure 2). We found no difference in absolute numbers or proportion of donor cells which were Foxp3+ in either the spleen or draining lymph nodes between the two experimental groups. This provides further evidence at an additional timepoint that high dose 4Y MBP immunization does not drive the development of a regulatory T cell phenotype.

We acknowledge that it would have been optimal to have ICS data of IL-10 in line with other cytokines, but technical challenges prevented us from obtaining robust ICS IL-10 staining which resulted in us taking this alternative approach. We have highlighted this limitation more clearly in the discussion.

6. Figure 5c like Figure 2b shows data from one experiment using pooled cells. To draw any conclusions regarding the biology, the authors need to present the combined data from all experiments.
Figure 5C is from a TCL so we feel the most appropriate way to present this data is from one experiment showing technical triplicates at each data point. We have included details in the legend which indicate that this result was representative for three independent experiments.

1. Ryan KR, McCue D, Anderton SM. Fas-mediated death and sensory adaptation limit the pathogenic potential of autoreactive T cells after strong antigenic stimulation. Journal of leukocyte biology. 2005;78(1):43-50.

Second Editorial Decision
18-Oct-2018

Dear Prof. Mellanby,

Thank you for submitting your revised manuscript ID eji.201847868.R1 entitled "PD-1 expression is upregulated on adapted T cells but is not required to maintain a hyporesponsive state" to the European Journal of Immunology. Your manuscript has been re-reviewed and the comments of the referees are included at the bottom of this letter.

Unfortunately, referee 2 was not satisfied with the revisions made and further major revision is requested.

Please note that the Executive Committee has pointed out that the conclusions in your revised manuscript have to be based on experiments that are reproducible and repeated at least one more time.

The journal does not encourage multiple rounds of revision and you should fully address the concerns of the referee in this final round of revision. Should you disagree with any of the referees' concerns, you should address this in your point-by-point response and provide solid scientific reasons for why you will not make the requested changes.

You should also pay close attention to the editorial comments included below. **In particular, please edit

your figure legends to follow Journal standards as outlined in the editorial comments. Please clearly state in all the Figure legends the number of sample/mice used and of independent experiments performed. Failure to do this will result in delays in the re-review process.**

Please note that submitting a revision of your manuscript does not guarantee eventual acceptance, and that your revision will be re-reviewed by the referees before a decision is rendered.

If the revision of the paper is expected to take more than three months, please inform the editorial office. Revisions taking longer than six months may be assessed by new referees to ensure the relevance and timeliness of the data.

Once again, thank you for submitting your manuscript to European Journal of Immunology and we look forward to receiving your revision.

Yours sincerely,
Ivan Shevchenko

On behalf of
Prof. Britta Engelhardt

Dr. Ivan Shevchenko
Editorial Office
European Journal of Immunology
e-mail: ejied@wiley.com

www.eji-journal.eu

Get the latest articles delivered directly to your desktop! Register now for the free Wiley Alerting Service at http://onlinelibrary.wiley.com/myprofile/alertManager

****

Reviewer: 1

Comments to the Author
All my comments were properly addressed.

Reviewer: 2

Comments to the Author
The authors have adequately addressed many of the issues raised previously. However, there are several remaining that were not fully addressed.

1. "The results shown in Figure 2b are from one single experiment with pooled spleens." Although I appreciate the difficulty is obtaining enough cells in these experiments to assess each mouse individually, it is still not appropriate to assume that one experiment with one sample per group provides sufficient evidence to support the authors' conclusions. I believe that the authors can address this deficiency in two ways: 1) remove the figure as suggested or 2) provide the collated or other replicate experiments in the supplementary information. Of the two, I would prefer #2 as that provides experimental transparency.

2. "Figure 3b shows the number of cells in the CNS. How have these numbers been calculated?" In this revision, the authors have added text, but that text does does not enable the reader to determine how the numbers were calculated. The reference provided (#43) does not mention any methodological detail about isolation, enumeration, or analysis of immune cells isolated from the CNS.

3. "The authors need to show their flow cytometric gating strategy." A gating strategy is supplied in Supplementary figure 1, which I assume is for the CNS immune cells. From this gating strategy, it is unclear how Tg4 CD4 T cells are distinguished from non-Tg4 CD4 T cells.

4. In response to the question about non-Tg4 cells, the authors responded, "Whist we acknowledge that other cells are present in the CNS at peak of disease, it is also important to acknowledge that the central drivers of the Tg4-B10.PLxC57BL/6 active EAE model are the
transferred T cells." The authors may be correct that these cells are the central initiators; however, initiation and propagation are two very different things. While the Tg4 CD4 T cells may be critical to initiation, it is the propagation of this targetted immune response that causes the damage and disease. Thus, if you are looking at the factors that are driving clinical disease, you cannot ignore the non-Tg4 cells. At the very least, their potential contribution to the disease process should be acknowledged.

5. "Figure 5c like Figure 2b shows data from one experiment using pooled cells." Like Figure 2b, this figure remains one representative experiment showing one pooled sample. This type of data presentation does not enable a robust assessment of the true effect size or reproducibility. Because these data are essential to the manuscript's conclusions, the authors need to either: 1) show the merged data from all three experiments or 2) provide the results from the other replicate experiments. It is sufficient is they are included in the Supplementary data; they just need to be accessible. As with Fig 2b, experimental

transparency is key.

Second Revision – authors' response

Dear Editor,

Many thanks for your recent email and for your patience whilst we ensure that all the concerns of the second reviewer have been adequately addressed.

Many thanks again for all your help and we look forward to hearing from you in due course.

Yours sincerely,

Richard Mellanby

Reviewer: 1

Comments to the Author
All my comments were properly addressed.

We are very pleased that we have properly addressed all of reviewer 1's comments.

Reviewer: 2

Comments to the Author
The authors have adequately addressed many of the issues raised previously. However, there are several remaining that were not fully addressed.

European Journal of Immunology

We are pleased that we have adequately addressed many of the issues raised previously and hope that the comments below ensure that the remaining concerns have been fully addressed.

1. "The results shown in Figure 2b are from one single experiment with pooled spleens." Although I appreciate the difficulty is obtaining enough cells in these experiments to assess each mouse individually, it is still not appropriate to assume that one experiment with one sample per group provides sufficient evidence to support the authors' conclusions. I believe that the authors can address this deficiency in two ways: 1) remove the figure as suggested or 2) provide the collated or other replicate experiments in the supplementary information. Of the two, I would prefer #2 as that provides experimental transparency.

In line with the request of the editor to minimise multiple submissions of revised text, we have decided to remove figure 2b. It does not alter any aspect of our conclusions and will hopefully resolve all concerns from the reviewer about this figure.

2. "Figure 3b shows the number of cells in the CNS. How have these numbers been calculated?" In this revision, the authors have added text, but that text does does not enable the reader to determine how the numbers were calculated. The reference provided (#43) does not mention any methodological detail about isolation, enumeration, or analysis of immune cells isolated from the CNS.

We have added the following text to the methods to explicitly describe how we isolated, enumerated and analysed immune cells isolated from the CNS. ‘Mice were sacrificed by $CO2$ asphyxiation and perfused with cold PBS. Spinal cords were removed by intrathecal hydrostatic pressure. Brain and spinal cords were cut into small pieces and digested with 2.5mg/ml collagenase (Worthington Biochemical) and 1mg/ml deoxyribonuclease (Sigma-Aldrich) for 25minutes at 37°C followed by mechanical disaggregation. Single cell suspensions were washed once in tissue culture medium. Mononuclear cells were retrieved from the interface of a 30:70% discontinuous Percoll gradient after centrifugation for 20 minutes at 850 x g. Cells were re-suspended in 1ml tissue culture medium and cell numbers were manually determined using a cell counting chamber, with the addition of 0.4% Trypan Blue for exclusion of dead cells. Analysis of CNS mononuclear phenotype and function was performed by flow cytometry as described in the relevant methods and results sections. The number of Tg4 donor cells was calculated based on the percentage of CD45.1+ cells of live singlet cells as determined by flow cytometric analysis.’

3. "The authors need to show their flow cytometric gating strategy." A gating strategy is supplied in Supplementary figure 1, which I assume is for the CNS immune cells. From this gating strategy, it is unclear how Tg4 CD4 T cells are distinguished from non-Tg4 CD4 T cells.

Supplementary figure 1 refers to the gating strategy to identify CD4+ TCL generated in vitro as stated in the text and is not relevant to the analysis of immune cells from the CNS. For the identification of immune cells in the CNS, a similar gating strategy was used as shown in supplementary figure 3 as stated in the text. The host and donor CD4+ cells are distinguishable by the expression of CD45.1 on the donor Tg4 cells. We have now expanded the legends of the supplementary figures to further clarify the gating strategies and donor cell identification:

'Supplementary figure 1 : Demonstration of gating strategy for the identification of CD4+ Tg4 TCL. Following in vitro culture of Tg4 splenocytes to obtain a Tg4 TCL, CD4+ T cells were identified by successively gating for cells, singlets, live cells, and CD4+ cells using appropriate markers.'

'Supplementary figure 2 : Demonstration of gating strategy for the identification of CD4+CD45.1+ Tg4 donor cells in the spleen of host CD45.2 mice at day 6 post immunization. CD4+ Tg4 donor cells were distinguished from host CD4+ T cells by surface expression of CD45.1.'

'Supplementary figure 3 : (A) Demonstration of gating strategy for the identification of donor CD4+CD45.1+ Tg4 T cells in the spleen of host CD45.2 mice at day 12 post immunization. CD4+ Tg4 donor cells were distinguished from host CD4+ T cells by surface expression of CD45.1. (B) Demonstration of gating strategy for identification of intracellular cytokine production following overnight stimulation with MBP.'

4. In response to the question about non-Tg4 cells, the authors responded, "Whist we acknowledge that other cells are present in the CNS at peak of disease, it is also important to acknowledge that the central drivers of the Tg4-B10.PLxC57BL/6 active EAE model are the transferred T cells." The authors may be correct that these cells are the central initiators; however, initiation and propagation are two very different things. While the Tg4 CD4 T cells may be critical to initiation, it is the propagation of this targetted immune response that causes the damage and disease. Thus, if you are looking at the factors that are driving clinical disease, you cannot ignore the non-Tg4 cells. At the very least, their potential

contribution to the disease process should be acknowledged.

We acknowledge the valid point made by reviewer 2, and we have now highlighted in the discussion the potential of non-Tg4 cells to contribute to the disease process. We have stated ‘In both groups, there were significant numbers of mononuclear cells in the CNS which were not Tg4 cells. Whilst the Tg4 CD4+ T cells are essential for initiating disease in B10.PLxC57BL/6 mice immunized with low dose MBP, non Tg4 immune cells are likely to play a role in propagating CNS pathology.’

5. "Figure 5c like Figure 2b shows data from one experiment using pooled cells." Like Figure 2b, this figure remains one representative experiment showing one pooled sample. This type of data presentation does not enable a robust assessment of the true effect size or reproducibility. Because these data are essential to the manuscript's conclusions, the authors need to either: 1) show the merged data from all three experiments or 2) provide the results from the other replicate experiments. It is sufficient is they are included in the Supplementary data; they just need to be accessible. As with Fig 2b, experimental transparency is key.

Whilst we appreciate that it is more favourable to be able to show single biological replicates over pooled data, the data originates from the same number of mice and is therefore more robust than if a single mouse was examined. We remain concerned that the reader will be perplexed why replicate data from one experiment is shown yet others are not. However, we are very keen to satisfy the concerns of the reviewer and so have included data from a second, independent experiment which confirms that a hyporesponsive phenotype can be generated in vitro in both Tg4 wildtype cells and Tg4 PD-1-/- cells in the main figures. We have measured proliferation by a second method (thymidine incorporation) to further demonstrate the robustness of our conclusion that PD-1 deficient T cells can acquire an adapted phenotype. By placing the data in the main figures, we hope this highlights very clearly the robustness and repeatability of our findings in a highly transparent fashion without the figure 5 block becoming unmanageably large.

Second Editorial Decision

09-Nov-2018

Dear Prof. Mellanby,

It is a pleasure to provisionally accept your manuscript entitled "PD-1 expression is upregulated on adapted T cells but is not required to maintain a hyporesponsive state" for publication in the European Journal of Immunology. For final acceptance, please follow the instructions below and return the requested items as soon as possible as we cannot process your manuscript further until all items listed below are dealt with.

Please note that EJI articles are now published online a few days after final acceptance (see Accepted Articles: https://onlinelibrary.wiley.com/toc/15214141/0/ja). The files used for the Accepted Articles are the final files and information supplied by you in Manuscript Central. You should therefore check that all the information (including author names) is correct as changes will NOT be permitted until the proofs stage.

We look forward to hearing from you and thank you for submitting your manuscript to the European Journal of Immunology.

Yours sincerely,
Ivan Shevchenko

on behalf of
Prof. Britta Engelhardt

Dr. Ivan Shevchenko
Editorial Office
European Journal of Immunology
e-mail: ejied@wiley.com
www.eji-journal.eu
